# Supplementary figures and images for: Impact and prognosis of the expression of IFN-α among tuberculosis patients
Source: PLoS One. 2020 Jul 15;15(7):e0235488. doi: 10.1371/journal.pone.0235488 (PMC7363073; doi:10.1371/journal.pone.0235488)

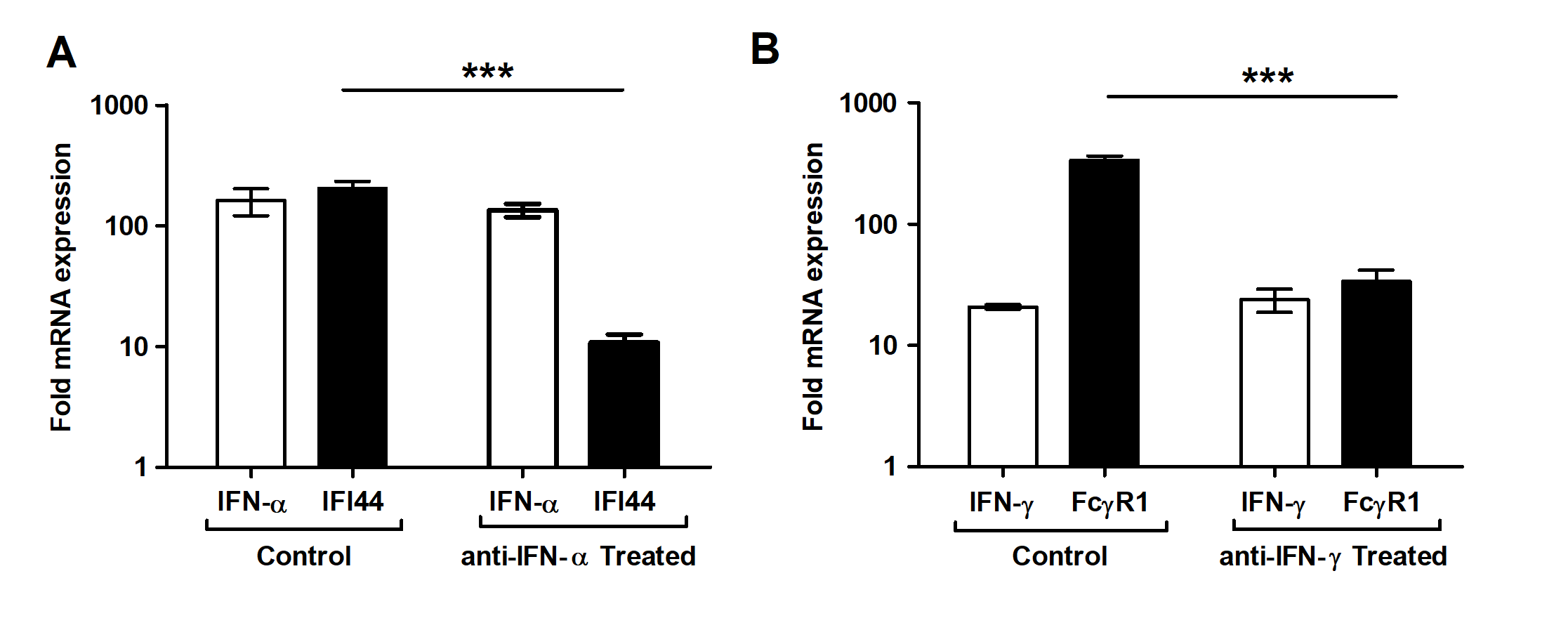

Supplement: S1 Fig — (Panel A) IFI44 (type I interferon inducible gene), and (Panel B) FcγR1 (type II interferon inducible gene) was monitored in PMA differentiated THP-1 cells. The gene expression was normalized with β-actin gene expression. The fold expression was calculated as described in methods. The bars represent the mean fold mRNA expression ± SD of three independent experiments. The data has been plotted in log10 scale. ***—p<0.0001, IFI44 and FcγR1 expression in control Vs cells treated with neutralizing anti-IFN-α and anti-IFN-γ antibodies respectively, Students t Test. (TIF) [file pone.0235488.s001.tif]

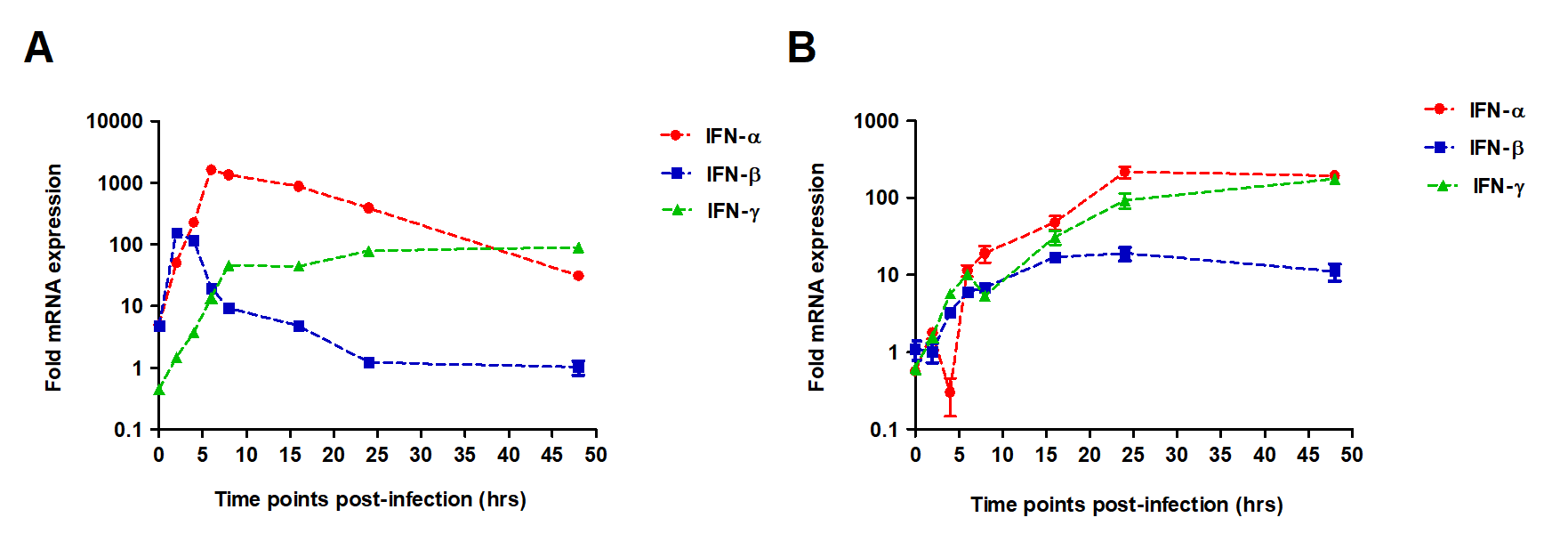

Supplement: S2 Fig — Time kinetics of mRNA expression as assessed by real-time PCR of type I and type II interferons in PMA-differentiated THP1 cells infected with M.tb (Panel A: Live; Panel B: Heat-killed). RNA was extracted at indicated time points and subjected to real-time PCR. Target gene expression was normalized with β-actin gene expression. The data has been calculated with the 2-ΔΔCt formula, as described in methods and has been plotted in log10 scale. I-Mean ± SD. (TIF) [file pone.0235488.s002.tif]
