# Supplementary material for: Impact and prognosis of the expression of IFN-α among tuberculosis patients
Source: PLoS One. 2020 Jul 15;15(7):e0235488. doi: 10.1371/journal.pone.0235488 (PMC7363073; doi:10.1371/journal.pone.0235488)
Supplement: Attachment — Submitted filename: Response to Reviewers-Vibha et al.docx [file pone.0235488.s004.docx]

**Comments to the Author:**

4. Is the manuscript presented in an intelligible fashion and written in Standard English?

PLOS ONE does not copyedit accepted manuscripts, so the language in submitted articles must be clear, correct, and unambiguous. Any typographical or grammatical errors should be corrected at revision, so please note any specific errors here.

Reviewer #1: No

Rebuttal: The manuscript has been revised for language and to improve the presentation.

**Review Comments to the Author:**

**Question:** The authors measure different IFNs by quantifying gene expression by RT-PCR and normalizing to β-actin and expressing relative values as 2^-ΔΔCT^. High expression was defined as mRNA expression of IFN >1 fold, however the results plotted show median values of less than 1 (Figure 1 & 2). There may be difference between the patient and controls for these values, but what does that mean if the fold expression is less than 1?

In parallel from the in vitro challenge model the fold expression is indeed much greater than 1, between 100-1000-fold. This makes the ex vivo data very challenging to interpret.

Rebuttal: As per the suggestion of reviewer #1, Figures 1 and 2 have been plotted on a log scale. The box-plots depict the range of fold mRNA expression of the 3 IFNs among TB patients (P) and household contacts (C). The ratio between: IFN Transcripts determined in an individual / Group mean of IFN Transcripts for Healthy Volunteers; established the ‘Fold’ expression for the IFN in the individual. Accordingly, in individuals wherein mRNA expression of an IFN was higher than the group mean of healthy volunteers (>1-fold) was considered as a high expression. Despite the wide ranging expression of IFN-α seen in patients and household contacts, the expression levels differed statistically. The median fold expression of IFN-α among the patients was 0.8 (25^th^ percentile - 0.2; 75^th^ percentile - 2.2) compared to 0.23 (25^th^ percentile - 0.1; 75^th^ percentile - 0.9) among contacts.

Among the 3 IFNs investigated alterations in the expression levels were limited to IFN-α and IFN-β among treated patients, not observed in case of IFN-γ. Despite low levels of expression (<1 fold) seen among a number of TB patients, it is the comparative analysis of the levels IFN-α expression detected at the baseline (untreated) with that estimated following completion of treatment is important, (Fig 2 and 3). Change in the levels IFN-α expression occurred following successful treatment and reduction in mycobacterial load. Further, wide ranging expression of IFN-α seen in patients is influenced by intrinsic features of the infecting strain (such as its virulence, its constituents, intracellular release of extracellular mycobacterial DNA (eDNA)) and host derived factors (such as levels of neutrophil response, circulating plasmacytoid Dendritic cells, PGE_2_ levels etc..).

While it is true that the *in vitro* expression in THP-1 cells is much higher (100-1000-fold) in comparison to that detected among TB patients and contacts, the reasons for this could be due to the fact that a homogenous population of *in vitro* differentiated macrophages were exposed to live virulent laboratory strain (H37Rv) of *M. tuberculosis* (MOI of 10:1). It has been shown that virulent strains of *M.tb* have the inherent capacity to stimulate vigorously several folds higher the production and secretion of Type I IFNs among human macrophages, (Novikov et al., *Mycobacterium tuberculosis* Triggers Host Type I IFN Signalling To Regulate IL-1b Production in Human Macrophages. J Immunol. 2011. 187:2540-2547). The functional utility of THP-1 cells in the study of mycobacterial infection has been extensively examined, (Mendoza-Coronel and Castanon-Arreola. Comparative evaluation of *in vitro* human macrophage models for mycobacterial infection study. FEMS. Pathogens and Disease, 2016, Vol. 74, No. 6. doi: 10.1093/femspd/ftw052).

**Question:** Related how was the house-keeping gene selected? Where there any differences in its expression between the different groups examined? Have the authors tested whether these modest gene expression differences reflect differences in circulating immune cell proportions?

Rebuttal: The median value for the 2^-ΔCt^ β-actin mRNA expression in three groups examined, i.e., patients, HHC and HV is 14.04, 14.23 and 14.39 respectively. This expression for the housekeeping gene, i.e., β-actin, in TB patients, household contacts and healthy volunteers did not differ significantly and therefore this has been used as a normalizing control. β-actin as a normalizing control has been used in our earlier studies, (Verma *et al*., Prevalence, Distribution and Functional Significance of the -237C to T Polymorphism in the IL-12Rb2 Promoter in Indian Tuberculosis Patients. PLOS ONE. 2012; 7(4): e34355).

We have not tested whether type I and type II interferons gene expression differences in tuberculosis reflect any differences in circulating immune cell proportions.

**Question:** Also, how do the authors reconcile their findings with the literature where although ISG signatures have been identified in TB disease by many groups, elevated IFN gene expression (Berry et al, Nature 2010) or more recently elevated protein levels (Llibre et al, Front Cell Infect Microbiol. 2019) have not been reported.

Rebuttal: Yes, it would have been ideal to be able to estimate both the specific mRNA and the corresponding protein namely IFN-α. There could be several reasons for this discrepancy such as: (1) IFN-α is a small molecular wt. protein with a short estimated half-life of 4 to 16hrs; (2) owing to receptor mediated endocytosis; or (3) degradation and inactivation by serum proteolytic activity, [Shechter *et al*., Prolonging the half-life of human interferon-α2 in Circulation: Design, preparation, and analysis of (2-sulfo-fluorenylmethoxycarbonyl) 7-Interferon-α. PNAS. January 30, 2001. Vol. 98, no. 3. 1212–1217]. In the present study we were unable to estimate IFN-α in serum using ELISA kits; the results were erratic and unreliable.

**Question:** How were patients defined to have TB disease?

Rebuttal: All patients underwent clinical examination, chest X-ray, sputum microscopy for acid fast bacilli (AFB), and routine laboratory tests. Diagnosis of tuberculosis was made on the basis of clinical and radiological assessment and detection of AFB in sputum. Scrutinizing clinical histories of patients, physical examination, and laboratory investigations ruled out the occurrence of concomitant intracellular infections, in the enrolled individuals. All patients were human immunodeficiency virus negative. For extra-pulmonary tuberculosis, patients who had organ specific signs and symptoms and microbiologically confirmed or clinically diagnosed case of TB involving organs other than lungs such as lymph nodes, pleural, bones, meninges, intestine, genitourinary tract etc. and/or constitutional symptoms like fever for more than 2 weeks, night sweats, significant weight loss etc. were included. The healthy family contacts and miscellaneous healthy laboratory volunteers were screened for clinical signs of tuberculosis and were radiologically examined; when warranted on the basis of the symptoms, additional tests such as sputum examination for AFB and erythrocyte sedimentation rate were undertaken.

**Question:** Which IFN-α subtype is detected with this assay?

Rebuttal: The primers used for detecting IFN-α mRNA were designed in such a way that they amplify IFN-α2, IFM-α6 and IFN-α14 (Verma *et al*., Prevalence, Distribution and Functional Significance of the -237C to T Polymorphism in the IL-12Rb2 Promoter in Indian Tuberculosis Patients. PLOS ONE. 2012. 7(4): e34355). The figure below showed the detailed sequence of all the subtypes with primer sequence highlighted in yellow.

Figure: Multiple sequence analysis showing different subtypes of IFN- α. The primer sequence has been highlighted in yellow. The nucleotide change has been shown and the dots represented the identical nucleotides in different subtypes.

**Question:** Figures 1 & 2 should be plotted with a log scale as the break in the axis is confusing.

Rebuttal: As suggested Figures 1 & 2 have been plotted on a log scale.

**Question:** Fig 4 should be plotted on log scale as many points are too close to the threshold, and the correlation plot should be included.

Rebuttal: Figure 4 has been plotted on log scale as suggested and the correlation plot has been included.

Figure 4: Scatter plot showing correlation between PGE2 levels and IFN-α mRNA expression. Higher the level of PGE2 lower is the expression of IFN-α and vice-versa. The spearman’s correlation constant is -0.563.

**Question:** How do the authors explain that blocked IFNα alone inhibits bacterial growth, but not when given with anti-IFNβ and anti-IFNγ? Do THP1 cells express IFNGR and IFNAR? To equal levels?

Rebuttal: Three	aliquots of *M.tb* infected THP-1 cells were individually exposed to	specific neutralizing antibodies for each IFN. Maximal inhibition of CFU was seen in cultures treated with antibodies specific for	IFN-α compared to cultures treated with anti-IFN-β and IFN-γ respectively. In cultures exposed to anti-IFN-α antibody, IFN-γ is able to exert its biological activity namely stimulate microbicidal activity resulting in the significant reduction in the enumerated CFU. This anti-mycobacterial activity was not hindered by the presence of IFN-β. In contrast in cultures exposed to anti-IFN-β antibody, both IFN-α and IFN-γ are present. However, the presence of IFN-α in the culture interferes with biological activity of IFN-γ. Earlier in our publication, Singhal et al., (Modulation of Gamma Interferon Receptor 1 by Mycobacterium tuberculosis: a Potential Immune Response Evasive Mechanism. Infection and Immunity. 2007. 75(5): 2500–2510), had shown the down regulation of IFNGR expression	during	infection with *M. tuberculosis*. IFN-α/β has been reported to down regulate the surface expression of IFNGR, by decreasing the mRNA expression of IFNGR (Rayamajhi et al., Induction of IFN-α/β enables Listeria monocytogenes to suppress macrophage activation by IFN-γ. J. Exp. Med. 2010. 207(2):327-337; Londino et al., Post-translational modification of the interferon-gamma receptor alters its stability and signaling. Biochem J. 2018. 474(20): 3543–3557.). The receptors IFNGR and IFNAR expression have been reported in THP1 cells on stimulation with PMA and in monocytes, respectively, (Londino *et al*., 2018; Kierney *et al*., Type I interferons down regulate myeloid cell IFNGR by inducing recruitment of an Egr3/Nab1 complex that silences *ifngr1* transcription1. J Immunol. 2013 September 15; 191(6): 3384–3392; Pogue *et al*., The Receptor for Type I IFNs Is Highly Expressed on Peripheral Blood B Cells and Monocytes and Mediates a Distinct Profile of Differentiation and Activation of These Cells. Journal of Interferon & Cytokine Research. 2004. 24:131–139).
